# Tetrathiomolybdate mediates cisplatin-induced p38 signaling and EGFR degradation and enhances response to cisplatin therapy in gynecologic cancers

**DOI:** 10.1038/srep15911

**Published:** 2015-11-16

**Authors:** Kyu Kwang Kim, Alex Han, Naohiro Yano, Jennifer R. Ribeiro, Elizabeth Lokich, Rakesh K. Singh, Richard G. Moore

**Affiliations:** 1 Molecular Therapeutics Laboratory, Program in Women's Oncology, Departments of Obstetrics and Gynecology, Women and Infants Hospital, Alpert Medical School, Brown University, Providence, RI, USA

## Abstract

Cisplatin and its analogs are among the most widely used chemotherapeutic agents against various types of cancer. It is known that cisplatin can activate epidermal growth factor receptor (EGFR), which may provide a survival benefit in cancers. Tetrathiomolybdate (TM) is a potent anti-cancer and anti-angiogenic agent and has been investigated in a number of clinical trials for cancer. In this study, we explore the therapeutic potential of TM on cisplatin-mediated EGFR regulation. Our study shows that TM is not cytotoxic, but exerts an anti-proliferative effect in ECC-1 cells. However, TM treatment prior to cisplatin markedly improves cisplatin-induced cytotoxicity. TM suppressed cisplatin-induced activation of EGFR while potentiating activation of p38; the activation of p38 signaling appeared to promote cisplatin-induced EGFR degradation. These results are in contrast to what we saw when cells were co-treated with cisplatin plus an EGFR tyrosine kinase inhibitor, where receptor activation was inhibited but receptor degradation was also blocked. Our current study is in agreement with previous findings that TM may have a therapeutic benefit by inhibiting EGFR activation. We furthermore provide evidence that TM may provide an additional benefit by potentiating p38 activation following cisplatin treatment, which may in turn promote receptor degradation by cisplatin.

Cisplatin, a DNA-intercalating platinum compound, and its analogs are widely used to treat human cancers and are some of the most effective agents available for treating cancers of the ovary, endometrium, head and neck, and lung. Cisplatin treatment is known to trigger diverse cellular responses including mitogen-activated protein kinase (MAPK) signaling pathways. MAPKs are serine/threonine protein kinases that consist of three distinct subgroups including p38 kinase, c-Jun N-terminal kinase (JNK), and extracellular signal-regulated kinase (ERK). p38 and JNK signaling regulate stress-mediated apoptosis, and their activation is known to be pivotal for cisplatin-induced cytotoxicity^1^. In contrast, ERK signaling is known to promote cancer cell survival, proliferation, and metastasis, and also contributes to cisplatin resistance^2^,^3^. Several studies have shown that blocking ERK signaling improves cisplatin sensitivity, suggesting its cytoprotective role against cisplatin treatment^4^,^5^,^6^. ERK is a downstream component of the epidermal growth factor receptor (EGFR)-Ras-Raf-MEK-ERK pathway. Activating mutations and/or overexpression of EGFR, Ras, or Raf are frequently found in human cancers^2^. As a result, much attention has focused on developing targeted anticancer therapies against this pathway^7^.

EGFR is a transmembrane receptor tyrosine kinase and belongs to the ErbB protein family that also includes ErbB2/HER2/Neu, ErbB3/HER3, and ErbB4/HER4. EGFR mutations, gene amplification, and overexpression have been found in a wide variety of human cancers, including those of the lung, ovary, head and neck, and breast^8^. EGFR overexpression is considered a poor prognostic marker^8^, and overexpression or elevated activity of EGFR are closely correlated with tumorigenesis and cancer progression^9^. EGFR overexpression also correlates with resistance against chemotherapy and radiation therapy^10^. EGFR tyrosine kinase inhibitors (TKIs) as single agents have shown potent clinical benefits in lung cancer patients harboring EGFR-activating mutations^11^. In addition, an anti-EGFR strategy was shown to enhance chemotherapy efficacy in an *in vitro* as well as an *in vivo* model of various cancers^12^,^13^. Importantly, cisplatin, as well as other DNA-targeting anti-cancer drugs including camptothecin and doxorubicin, was shown to induce EGFR tyrosine phosphorylation, and its blockage with the EGFR TKI AG1478 enhanced cisplatin-induced cell death in human glioma cells^14^. Similarly, a synergistic interaction between cisplatin and the EGFR TKI gefitinib was found in lung cancer^15^. In addition, it has been reported that nuclear localization of EGFR contributes significantly to DNA damage repair after cisplatin treatment^10^, suggesting that combination treatment with anti-EGFR therapies and cisplatin would be therapeutically beneficial.

Apart from approaches to block EGFR activation, promoting its degradation is also viewed as an attractive strategy for anticancer therapies. EGFR degradation is a major desensitization process that can prevent receptor hyperactivation commonly found in cancer. EGFR degradation was shown to play a crucial role in cisplatin sensitivity^16^. The precise mechanism(s) of EGFR regulation involving receptor internalization and degradation remains to be elucidated. In general, EGF stimulation mediates phosphorylation of multiple tyrosine sites including Y1045, which provides a docking site for the E3 ubiquitin-ligase c-Cbl, followed by receptor ubiquitination, internalization, and endosomal sorting, ultimately leading to receptor degradation in lysosomes^17^. Ahsan *et al.* found that cisplatin treatment, like EGF stimulation, also mediates EGFR tyrosine phosphorylation on Y1045 and promotes receptor ubiquitination and degradation^16^. Interestingly, accumulating evidence suggests that p38 signaling also plays a crucial role in EGFR regulation^18^,^19^. p38-dependent phosphorylation of EGFR serine residues, as opposed to the tyrosine site Y1045, was responsible for receptor internalization and degradation^20^,^21^,^22^. Yet, it remains unclear if cisplatin treatment involves p38 signaling for receptor degradation.

Tetrathiomolybdate (TM) is an effective non-toxic copper-binding agent initially developed to treat copper overload disorders observed in Wilson’s disease. Several studies have demonstrated that TM also possesses potent anti-cancer and anti-angiogenic potential against various types of cancer^23^,^24^ and has been evaluated in several clinical trials for cancer. TM is known to target various copper-dependent enzymes including copper/zinc superoxide dismutase (SOD1)^25^, lysyl oxidase (LOX)^26^, and cytochrome c oxidase (CCO)^25^,^27^. Among these enzymes, SOD1 has been identified as a main therapeutic target of TM for its anti-proliferative and anti-angiogenic effects^28^. Interestingly, TM was found to abolish VEGF- or FGF-2-mediated activation of ERK signaling^28^. In addition, growth factor-induced activation of EGFR or IGF-1Rβ was attenuated by TM^29^. Recently, our studies have documented that TM treatment potentiates cytotoxic effects of doxorubicin or other chemotherapeutic agents in endometrial and ovarian cancer cells^30^,^31^. We also found that TM in combination with doxorubicin induces a drastic increase in p38 activation^30^,^31^. In the present study, we tested the hypothesis that TM also potentiates cisplatin sensitivity in a panel of gynecologic cancer cells. Specifically, we evaluated the therapeutic potential of TM to mediate cisplatin sensitivity in ECC-1 endometrial cancer cells by exploring two distinct EGFR desensitization pathways: the suppression of receptor activation and potentiation of receptor degradation. We also sought to address the role of p38 signaling in EGFR regulation upon cisplatin exposure.

## Results

### TM inhibits cell proliferation and sensitizes cancer cells to cisplatin

**Figure 1:**
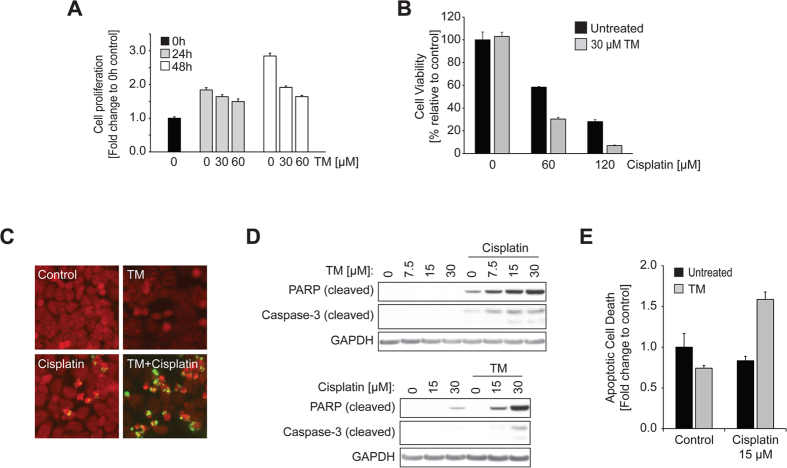
TM inhibits cell proliferation and sensitizes cancer cells to cisplatin. (**A**) ECC-1 cells were treated with TM (0, 30, 60 μM) for the indicated times, after which the cells were fixed, and the cell proliferation was measured by SRB assay. (**B**) ECC-1 cells were incubated with or without TM (30 μM) for 24 h, after which the cells were treated with cisplatin (0, 60, 120 μM) for another 24 h. The cell viability was evaluated using MTS assay. (**C**) ECC-1 cells were incubated with or without TM (30 μM) for 18 h, after which the cells were incubated with or without cisplatin (30 μM) for another 24 h. The apoptotic cells were visualized by the TUNEL method that detects DNA fragmentations in apoptotic cells. (**D**) Top: ECC-1 cells were treated with or without TM for 24 h at the indicated concentrations, followed by cisplatin treatment (30 μM) for 24 h. Bottom: ECC-1 cells were incubated with or without TM (30 μM) for 24 h, after which the cells were treated with cisplatin at the indicated concentrations for 24 h. The levels of cleaved PARP, cleaved caspase-3, or GAPDH were determined by Western blot analysis. (**E**) ECC-1 cells were incubated with TM (30 μM) for 24 h, after which each cell line was treated with cisplatin (15 μM) for 24 h. Apoptotic cell death was determined by caspase-3/7 activity.

Anti-proliferative effects of TM have been reported^28^,^29^,^32^. To verify these results, we treated ECC-1 human endometrial cancer cells with various concentrations of TM (0, 30, 60 μM) and evaluated the effect of TM on cell proliferation (Fig. 1A). After treatment for 24 or 48 h, the cell population was analyzed using SRB cell proliferation assay and compared to that of the untreated cells before treatment (0 h). We found that TM inhibits ECC-1 cell proliferation in a dose- and time-dependent manner. Cells incubated with TM for 24 h at 30 μM showed only moderate inhibition (10.9%) compared to the untreated. Anti-proliferative effects of TM (30 μM) were further increased (32.7%) after 48 h (Fig. 1A). Next, we interrogated if TM potentiates cisplatin sensitivity in ECC-1 cells using an MTS cell viability assay. This assay revealed that the viability of cells treated with both TM and cisplatin was reduced compared to cells exposed to cisplatin or TM alone (Fig. 1B). A common method for visualizing cellular apoptotic events is the TUNEL assay. We observed a clear appearance of TUNEL-positive cells (labeled FITC) after cisplatin treatment (Fig. 1C). The number of apoptotic events was substantially increased when cells were pretreated with TM followed by cisplatin treatment while TM treatment alone did not cause apoptosis in ECC-1 cells. Next, we performed immunoblotting to detect the activation of caspase-3 and the cleavage of PARP, characteristic markers for the induction of apoptosis. As shown in Fig. 1D (top panel), TM pretreatment (24 h) prior to cisplatin treatment (30 μM, 24 h) clearly increased cellular apoptosis in ECC-1 cells, compared to those only exposed to cisplatin. TM-mediated cisplatin sensitivity was found to be dose-dependent. Similarly, a fixed concentration of TM (30 μM) caused a dose-dependent increase in cisplatin-mediated cytotoxicity (0, 15, 30 μM) in ECC-1 cells (bottom panel). In order to measure the effects of drugs on apoptosis quantitatively we carried out the caspase-3/7 activity assay. This assay revealed that ECC-1 cells exposed to combinatorial treatment had a 1.9-fold increase in apoptosis over cells exposed to cisplatin alone (Fig. 1E).

### The inhibition of SOD1 by TM suppresses cisplatin-induced activation of ERK.

**Figure 2:**
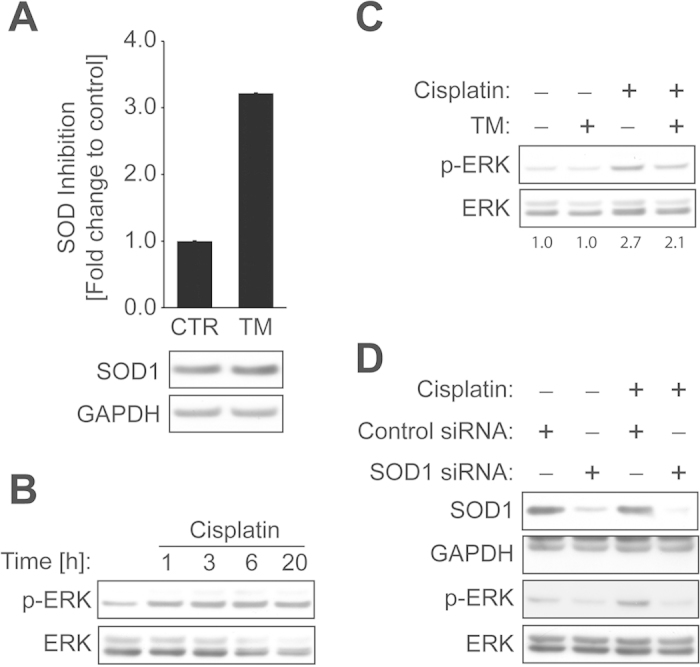
The inhibition of SOD1 suppresses cisplatin-induced activation of ERK. (**A**) Top: ECC-1 cells were treated with or without TM (15 μM) for 20 h. SOD activity was measured as described in Materials and Methods. Bottom: ECC-1 cells were incubated with or without TM (30 μM) for 24 h. The expression of SOD1 or GAPDH was measured by Western blot analysis. (**B**) ECC-1 cells were treated with cisplatin (30 μM) for the indicated times. The levels of p-ERK or ERK were determined by Western blot analysis. (**C**) ECC-1 cells were pretreated with TM (30 μM) for 24 h, followed by treatment with or without cisplatin (30 μM) for 8.5 h. The lysates were subjected to Western blot analysis for the detection of p-ERK or ERK expression. Band ratio (p-ERK/ERK) was determined by densitometry. (**D**) ECC-1 cells were transfected for 42 h with 50 nM siRNA pool against SOD1 or with non-targeting control, after which the cells were treated with or without cisplatin (30 μM) for 3 h. The lysates were subjected to Western blot analysis for SOD1, GAPDH (lower band), p-ERK, or ERK.

SOD1 is a proposed therapeutic target of TM^28^. Thus, we tested the effect of TM on SOD activity in our cell line model. ECC-1 cells incubated with TM (15 μM, 20 h) showed a significant reduction in SOD activity (Fig. 2A Top panel). To clarify that this finding was not a result of TM-mediated alteration of SOD1 protein levels, immunoblot analysis was carried out. Our data revealed that TM, even at a concentration 2-fold higher (30 μM, 24 h) than what was used for the SOD activity assay, did not alter the protein levels of SOD1 in ECC-1 cells (Fig. 2A Bottom panel). TM treatment has been reported to attenuate growth factor-induced ERK activation through SOD1 inhibition^29^. Activation of ERK signaling may play a prosurvival function in cancer cells upon cisplatin exposure^4^. Thus, we sought to investigate if cisplatin activates ERK signaling in ECC-1 cells, and if TM treatment blocks this activation. We found that cisplatin induces ERK phosphorylation in ECC-1 cells (Fig. 2B). TM treatment or siRNA-mediated SOD1 knockdown clearly inhibits cisplatin-induced ERK phosphorylation (Fig. 2C,D), suggesting that TM-mediated SOD1 inhibition suppresses cisplatin-induced ERK activation.

### TM inhibits cisplatin-mediated EGFR tyrosine phosphorylation

**Figure 3:**
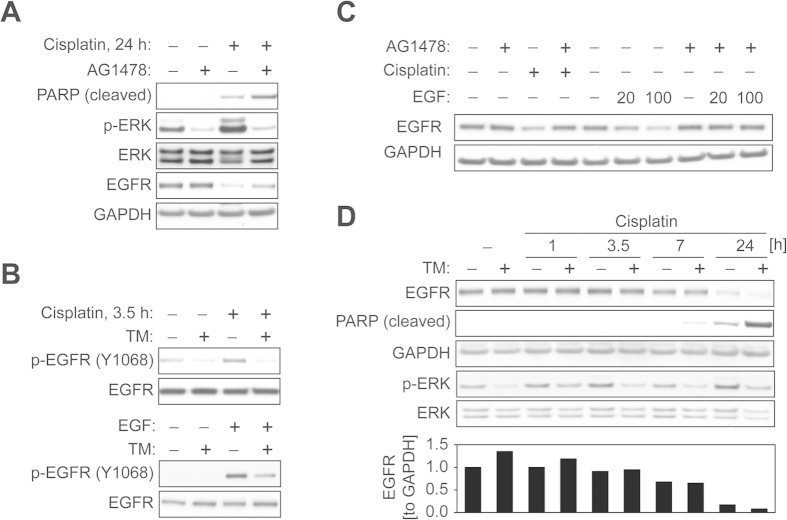
TM inhibits cisplatin-mediated EGFR tyrosine phosphorylation. (**A**) ECC-1 cells were treated with cisplatin (30 μM) for 24 h with or without AG1478 (EGFR inhibitor, 5 μM, 30 min pretreatment). The expression of cleaved PARP, p-ERK, ERK or GAPDH and downregulation of EGFR caused by cisplatin were assessed by Western blot analysis. (**B**) ECC-1 cells were treated with TM (30 μM) for 48 h, and then exposed to either cisplatin (30 μM, 3.5 h) or EGF (33 ng/ml, 10 min). The phosphorylation of EGFR Tyr-1068 or total EGFR was analyzed by Western blotting. (**C**) ECC-1 cells were treated with cisplatin (120 μM, 6 h) or exposed to EGF (20 or 100 ng/ml) for a prolonged duration (3 h) in the absence or presence of 5 μM of AG1478. The changes in EGFR expression were analyzed by Western blotting. (**D**) Top: ECC-1 cells were treated with or without TM (30 μM) for 48 h, after which the cells were exposed to cisplatin (30 μM) for the indicated times. The levels of the indicated proteins were measured by Western blot analysis. Bottom: The levels of EGFR expression (relative to GAPDH) were calculated using densitometry analysis.

To delineate the molecular mechanism of cisplatin-induced ERK activation in ECC-1 cells, we examined EGFR activation, which occurs upstream of ERK signaling. Here, our data show that blockage of EGFR with the ATP-competitive TKI AG1478 sensitizes ECC-1 cells to cisplatin treatment, leading to an increase in cellular apoptosis as indicated by an increase in PARP cleavage (Fig. 3A). Benhar *et al.* reported that cisplatin activates EGFR that lacks its extracellular domain^14^, suggesting that cisplatin-mediated EGFR activation may involve molecular mechanisms that are distinct from ones triggered by a physiological ligand. Thus, we sought to determine if TM suppresses cisplatin-induced EGFR activation. Cisplatin treatment in ECC-1 cells induced tyrosine phosphorylation of EGFR at Y1068, which is involved in signal transduction to ERK^33^. The level of EGFR phosphorylation by both EGF and cisplatin was decreased in cells pretreated with TM (Fig. 3B), suggesting that the effects of TM on EGFR deactivation occur regardless of the type of stimulus. Tyrosine phosphorylation of EGFR results in receptor ubiquitination, internalization, and endosomal sorting, ultimately leading to its degradation in lysosomes^17^. We found that treatment with AG1478 clearly inhibited EGFR downregulation induced by both cisplatin and EGF stimulation (Fig. 3C). Next, we examined the effects of TM on EGFR protein levels. ECC-1 cells pretreated with vehicle or TM were incubated with cisplatin. Cisplatin treatment for 7 h caused a slight downregulation of EGFR levels, which was further enhanced after 24 h (Fig. 3D). Interestingly, our data show that TM treatment, unlike our observations with AG1478 treatment (Fig. 3C), did not restore EGFR expression. The levels of EGFR in cells treated with TM and cisplatin in combination were comparable to those in cells treated with cisplatin alone for 7 h, and were further reduced after 24 h combinatorial treatment (Fig. 3D). This effect on EGFR levels occurred simultaneously with blockage of ERK activation, and correlated with an increase in cleaved PARP.

### Cisplatin-mediated p38 activation is involved in EGFR degradation

**Figure 4:**
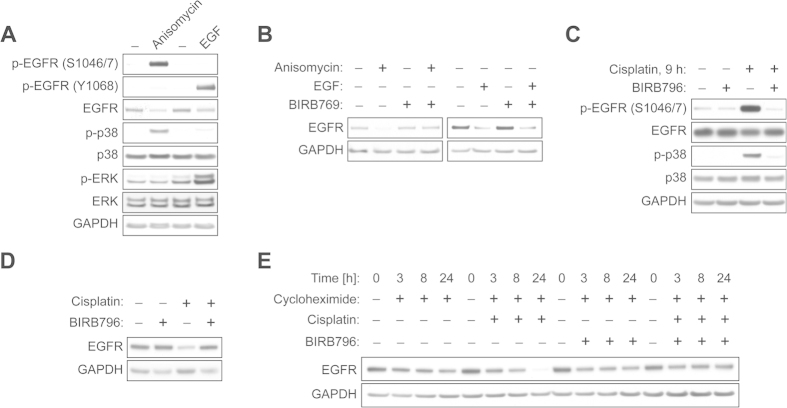
Cisplatin-mediated p38 activation causes EGFR degradation. (**A**) ECC-1 cells were exposed to vehicle, anisomycin (30 μM, 1 h) or EGF (100 ng/ml, 10 min). The activation of p38 or ERK was evaluated by Western blot analysis using antibodies as indicated. EGFR phosphorylation was analyzed by site-specific p-EGFR antibodies against Tyr-1068 or Ser-1046/7. (**B**) ECC-1 cells were treated with vehicle, EGF (100 ng/ml, 10 min) or anisomycin (30 μM, 1 h) with or without BIRB769 pretreatment (1 μM, 30 min). The levels of EGFR or GAPDH were measured by Western blot analysis. (**C**) ECC-1 cells were incubated with vehicle or cisplatin (30 μM) for 9 h with or without BIRB796 (1 μM, 30 min pretreatment). The lysates were prepared and subjected to Western blotting for p-EGFR (Ser-1046/7), EGFR, p-p38, p38 or GAPDH expression. (**D**) ECC-1 cells were exposed to vehicle or cisplatin (120 μM) for 6 h with or without BIRB769 (1 μM). Immunoblotting was performed to measure levels of EGFR or GAPDH. (E) ECC-1 cells were treated with vehicle, cisplatin (30 μM), and BIRB769 (1 μM) alone or in combination in the presence of cycloheximide (25 μM). Cells were collected at indicated times and subjected to Western blotting for EGFR or GAPDH.

On the basis of our initial findings described above, we reasoned that there might be an alternative route through which TM maintains EGFR downregulation by cisplatin. One possible candidate to negatively regulate EGFR levels is p38 kinase^18^. In order to determine if cisplatin causes p38-dependent degradation of EGFR, we first tested the effects of anisomycin, a potent p38-inducing agent, on EGFR protein levels in ECC-1 cells. As shown (Fig. 4A), anisomycin clearly induced p38 activation, leading to phosphorylation of EGFR at serine sites (S1046/7) predominantly over a tyrosine site (Y1068), and caused marked EGFR downregulation. In contrast, EGF stimulation of ECC-1 cells caused tyrosine phosphorylation of EGFR (Y1068), activation of the downstream target ERK, and subsequent EGFR downregulation. The blockage of p38 signaling using BIRB769, a pharmacologic p38 inhibitor, restored EGFR expression downregulated by anisomycin but not by EGF stimulation (Fig. 4B). Next, we sought to explore if cisplatin also targets EGFR though p38 signaling. We found that cisplatin treatment (30 μM) for 9 h caused potent activation of p38 and induced serine phosphorylation of EGFR in ECC-1 cells, which was impeded by p38 inhibition using BIRB769 (Fig. 4C). We then employed a higher dose of cisplatin (120 μM) for a shorter duration (6 h) and monitored receptor levels. This condition was sufficient to induce clear EGFR downregulation (Fig. 4D). The blockage of p38 using BIRB769 markedly restored EGFR expression, suggesting that activation of p38 signaling is one mechanism of cisplatin-mediated EGFR downregulation. To further clarify this hypothesis, we inhibited EGFR protein synthesis using cycloheximide and evaluated EGFR expression after cisplatin treatment with or without p38 inhibition. As shown in Fig. 4E, EGFR was downregulated more rapidly in cells cotreated with cycloheximide and cisplatin compared to those only exposed to cycloheximide treatment. We also found that pharmacologic inhibition of p38 almost completely blocks cisplatin-induced EGFR downregulation, further supporting that cisplatin promotes EGFR degradation via p38 signaling.

### TM pretreatment potentiates cisplatin-mediated p38 activation and EGFR serine phosphorylation

**Figure 5:**
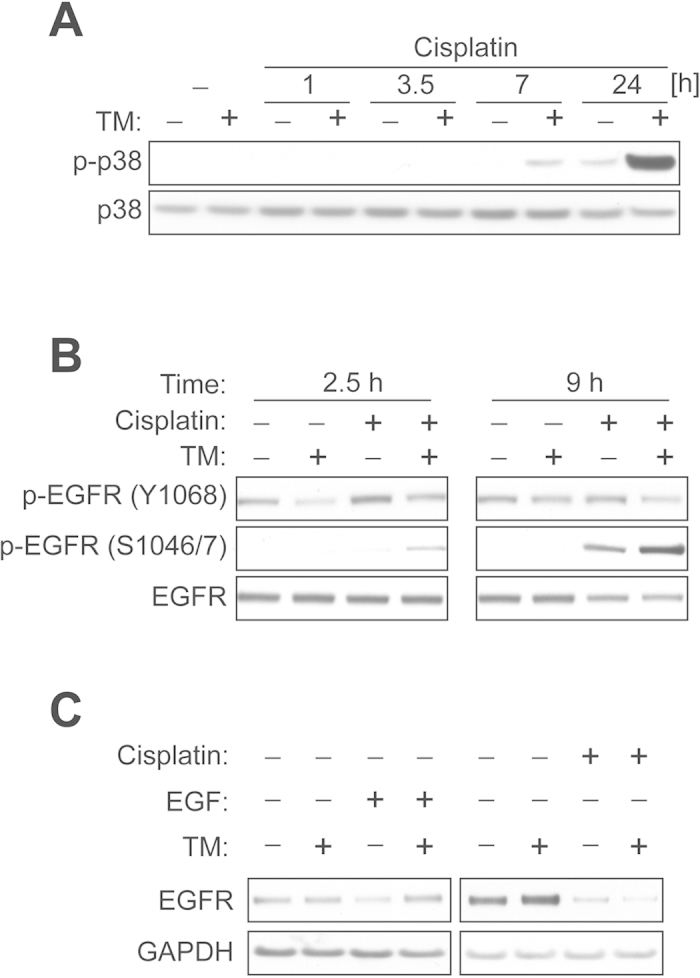
TM pretreatment potentiates cisplatin-mediated p38 activation and EGFR serine phosphorylation. (**A**) ECC-1 cells were treated with or without TM (30 μM) for 48 h, after which the cells were exposed to cisplatin (30 μM) for the indicated times. The levels of p-p38 or p38 were measured by Western blot analysis. (**B**) ECC-1 cells were treated with or without TM (30 μM) for 48 h, after which the cells were exposed to vehicle or cisplatin (30 μM) for the indicated times. EGFR phosphorylation was determined by Western blot analysis using site specific antibodies for Tyr-1068 or Ser-1046/7. (**C**) ECC-1 cells were treated with vehicle, EGF (20 ng/ml, 3 h) or cisplatin (120 μM, 6 h) with or without TM pretreatment (30 μM, 48 h). The levels of EGFR or GAPDH were measured by Western blot analysis.

Our previous studies have reported that TM treatment in combination with doxorubicin mediates p38 activation in cancer cells^30^,^31^. Thus, we investigated if TM pretreatment potentiates the activation of p38 caused by cisplatin treatment. As shown (Fig. 5A), cells treated with both TM and cisplatin (7 or 24 h post-treatment) showed a substantial increase in p38 activation compared to those only exposed to cisplatin for the same duration. Interestingly, we found that siRNA-mediated SOD1 knockdown in ECC-1 cells did not potentiate cisplatin-mediated p38 activation (Supplementary Fig. S1), suggesting that TM might regulate p38 by a mechanism independent of SOD1. Next, we investigated if TM potentiates EGFR serine phosphorylation following cisplatin treatment. As expected, cisplatin treatment in ECC-1 cells for 2.5 h clearly induced EGFR tyrosine phosphorylation on Y1068 (Fig. 5B). We observed that TM pretreatment diminishes tyrosine phosphorylation by cisplatin, yet causes the appearance of receptor serine phosphorylation (S1046/7) when compared to cells only exposed to cisplatin for the same duration (2.5 h). The effect of TM on serine phosphorylation was further pronounced after 9 h cisplatin exposure.

EGF stimulation involves EGFR tyrosine phosphorylation for receptor degradation. In contrast, our present findings suggest that cisplatin mediates receptor degradation in part through p38 signaling, which can be potentiated by TM treatment (Fig. 5A). Therefore, we sought to investigate if TM regulates EGFR degradation differently depending on the type of stimulus. As shown (Fig. 5C), TM pretreatment blocked EGFR degradation induced by EGF stimulation, possibly through suppression of tyrosine phosphorylation. In contrast, the receptor degradation mediated by cisplatin was not blocked by TM treatment, suggesting that treatment with TM may provide a unique therapeutic benefit by inhibiting receptor activation and promoting receptor degradation through p38 signaling.

### Therapeutic implications of TM-mediated p38 activation.

**Figure 6:**
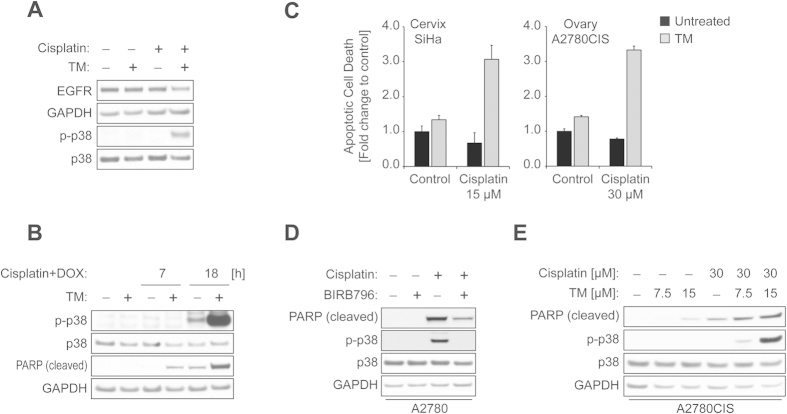
Therapeutic implications of TM-mediated p38 activation. (**A**) ECC-1 cells treated with vehicle or TM (30 μM) for 48 h were exposed to vehicle or cisplatin (120 μM) for 1 h. The cells were then washed with PBS, replenished with drug-free medium, and further incubated for 18 h. The levels of EGFR, GAPDH, p-p38, or p38 were measured by Western blot analysis. (**B**) ECC-1 cells pretreated with vehicle or TM (30 μM, 50 h) were exposed to combination therapy of cisplatin (8 μM) and doxorubicin (DOX, 5 μM) for indicated times. The lysates were subjected to Western blot analysis for p-p38, p38, cleaved PARP or GAPDH expression. (**C**) Indicated cell lines were incubated with TM (30 μM) for 24 h, after which each cell line was treated with cisplatin for 18 h at the indicated concentrations. Apoptotic cell death was determined by caspase-3/7 activity. (**D**) The lysates from A2780 cells treated with vehicle or cisplatin (60 μM) for 11 h in the absence or presence of BIRB769 (1 μM, 30 min pretreatment) were subjected to Western blot analysis for indicated proteins. (**E**) A2780CIS cells were treated with vehicle or TM at indicated doses for 18 h, after which the cells were treated with or without cisplatin (30 μM) for 27 h. The lysates were collected and subjected to Western blotting for cleaved PARP, p-p38, p38, or GAPDH.

Plasma concentrations of cisplatin decrease rapidly with a half-life of fewer than 30 minutes^34^. To evaluate the effects of cisplatin on EGFR regulation following short-term exposure, cells pretreated with or without TM were then incubated with cisplatin (120 μM) for 1 h, washed, and replenished with drug-free medium for 18 h before the levels of EGFR were examined. We found that cisplatin treatment alone under this condition was not sufficient to mediate receptor downregulation in ECC-1 cells. However, the level of EGFR was clearly decreased when cells were treated with both TM and cisplatin, which was accompanied by the appearance of p38 activation (Fig. 6A), suggesting that TM-induced p38 potentiation may trigger receptor degradation following short-term cisplatin exposure.

Next, to evaluate some clinical implications of TM treatment, we tested TM with the combination of cisplatin and doxorubicin, which has been used in patients with advanced or recurrent endometrial carcinoma^35^. Concentrations of cisplatin (8 μM) and doxorubicin (5 μM) that were used to treat cells here were chosen based on the treatment doses used. The ratio of cisplatin to doxorubicin used clinically (50 mg/m^2^ cisplatin and 60 mg/m^2^ doxorubicin, ratio: 0.83) was preserved^35^. Here, we found that TM markedly increased p38 activation in cells treated with cisplatin and doxorubicin in combination, compared to cells treated with the combination therapy alone. Additionally, TM treatment enhanced cell death induced by the combination therapy (cleaved PARP, Fig. 6B), suggesting a potential benefit of TM as an adjuvant to therapy.

To determine the potential relevance of these findings in other gynecological cancer cells, we used the caspase-3/7 activity assay to test the effects of TM on cisplatin sensitivity in SiHa cervical and cisplatin-resistant A2780CIS ovarian cancer cells. Similar to what we observed in ECC-1 cells (Fig. 1E), we saw that SiHa and A2780CIS cells exposed to the combination treatment of TM and cisplatin showed a respective 4.6- or 4.3-fold increase in apoptosis compared to those only treated with cisplatin (Fig. 6C). These results suggest that TM may be an effective adjuvant therapy in multiple tumor types, including those that are resistant to cisplatin. In support of this hypothesis, insufficient activation of p38 signaling was implicated in a failure to respond to cisplatin treatment in resistant cancer cells^1^. Thus, we sought to interrogate if TM-mediated p38 potentiation sensitizes resistant cells to cisplatin. To this end, we employed the cisplatin-resistant A2780CIS ovarian cell line and its isogenic cisplatin-sensitive counterpart A2780. Our initial data indicated that A2780CIS cells are more resistant to cisplatin compared to A2780 cells (Data not shown), which agrees with the previous report^36^. We also observed that blockage of p38 signaling in cisplatin-sensitive A2780 cells caused the inhibition of cisplatin-induced cytotoxicity (Fig. 6D), suggesting a proapoptotic role for p38 in these cells. Next, A2780CIS cells pretreated with vehicle or TM (18 h) were then exposed to vehicle or cisplatin (30 μM) for 27 h. Immunoblotting revealed that TM and cisplatin in combination induce a marked increase in p38 activation in A2780CIS cells, compared to those treated with TM or cisplatin as single agents. This increase in p-p38 correlated with an increase in cell death (Fig. 6C). Taken together, our data here suggest that TM may possess a therapeutic benefit by potentiating p38 activation for the treatment of cancer.

## Discussion

In this study, we showed that TM potentiates cisplatin-induced cytotoxicity in a panel of gynecologic cancer cell lines. TM as a single agent was not cytotoxic (Fig. 1C,D), and only showed dose- and time-dependent anti-proliferative effects in ECC-1 cells (Fig. 1A). However, TM prior to cisplatin treatment was found to significantly improve cisplatin-induced cytotoxicity (Fig. 1C,D,E). We found that TM pretreatment suppresses cisplatin-induced activation of prosurvival EGFR and ERK signaling, while potentiating the activation of proapoptotic p38 signaling caused by cisplatin. We also found that p38 activation is one mechanism of cisplatin-induced EGFR degradation. Therefore, TM-mediated potentiation of p38 activation may provide a unique therapeutic benefit in EGFR regulation. Unlike a conventional EGFR TKI that inhibits receptor activity but also blocks EGFR degradation (Fig. 3C), treatment with TM in combination with cisplatin inhibits EGFR activation by cisplatin without blocking receptor degradation. Such a dual attack on EGFR signaling—blocking receptor activation and maintaining receptor degradation—may confer an additional advantage over traditional EGFR TKI therapies. It has been suggested that SOD1 is the main therapeutic target of TM that executes its anti-proliferative and anti-angiogenic effects^28^. In our study, TM-induced SOD1 inhibition was responsible for a reduction in cisplatin-induced ERK activation. Phosphorylation of proteins is controlled by the opposing effects of kinase and phosphatase activities. Tyrosine phosphatase is known to be regulated by a redox mechanism. Previous studies by Juarez *et al.*^29^ proposed that TM-mediated SOD1 inhibition alters cellular redox status, activates protein-tyrosine phosphatase 1B, which is known to inactivate or dephosphorylate receptor tyrosine kinases such as EGFR, and leads to the downregulation of ERK phosphorylation upon growth factor stimulation. Other mechanisms in TM-mediated inhibition of ERK activation cannot be ruled out.

Accumulating evidence suggests that p38 signaling plays a significant role in mediating EGFR regulation. Cisplatin was shown to cause EGFR threonine phosphorylation and receptor internalization in a p38-dependent manner, and pharmacologic inhibition of p38, but not JNK or ERK, suppressed cisplatin-induced EGFR phosphorylation^37^. Cisplatin was also found to induce p38-dependent receptor internalization in SW480 cells^19^. It is noteworthy that these cells express high levels of EGFR and rely on autocrine TGF-α, an EGFR ligand, for survival. Thus, blocking receptor internalization reduced the cytotoxicity of cisplatin, suggesting that p38 activation and subsequent receptor internalization play a crucial role in augmenting the efficacy of cisplatin and, possibly, other chemotherapeutic agents that employ p38 activation^19^. Cancer cells with acquired cisplatin resistance were shown to express higher levels of EGFR, compared to their parental cell lines, and were more dependent on EGFR signaling^38^. In addition, insufficient activation of p38 following cisplatin treatment can hamper the efficacy of cisplatin in resistant cancer cells^1^. Thus, TM treatment, through the potentiation of p38 activation, may trigger EGFR internalization and/or degradation, which in turn may sensitize drug-resistant cancer cells to chemotherapy such as cisplatin.

EGFR degradation is considered a major desensitization process following receptor activation.

EGFR TKIs block EGFR activation, but also its subsequent degradation upon cisplatin exposure. Our data here and others’^14^ suggest that EGFR TKI treatment potentiates cisplatin-induced cytotoxicity in various cancer types. However, Ahsan *et al.* reported that blocking EGFR degradation by EGFR TKI causes a reduction in cisplatin-induced cell death^16^. This exemplifies the complexity of cellular EGFR functions and also supports an anti-EGFR strategy that promotes both EGFR desensitization pathways: EGFR degradation and inhibition of EGFR tyrosine kinase activity. TM inhibits cisplatin-induced EGFR tyrosine phosphorylation (Fig. 3B), but, unlike EGFR TKIs, also promotes receptor degradation (Fig. 5C). EGFR degradation may provide a therapeutic benefit by disrupting its interactions with other proteins. EGFR interacts with its family members, or with other receptor proteins such as integrin and urokinase receptor^39^,^40^. EGFR removal by TM in combination with chemotherapy could serve to downregulate such interactions. Moreover, EGFR is known to elicit prosurvival functions by employing a kinase activity-independent mechanism^41^,^42^. In such instances, EGFR degradation by TM could achieve an additional therapeutic effect over a traditional TKI, which would only target receptor activation.

The *in vivo* potential of TM treatment is illustrated in an elegant study by Ishida *et al.* employing the K14-HPV16/E_2_ transgenic mouse model of spontaneous cervical cancer, which showed that TM addition increases cisplatin-DNA adducts in tumors, but not in normal organs. TM also enhanced cisplatin efficacy in these mice^43^. It is possible that TM-mediated enhancement of cisplatin-DNA adducts contributes to potentiation of stress-responsive pathways such as p38 signaling. In addition, dual specificity phosphatase-1 (DUSP-1) is the MAPK phosphatase that targets p38. However, our preliminary study suggested that TM prior to cisplatin treatment may not alter DUSP-1 expression in ECC-1 cells compared to that of cells only exposed to cisplatin (Data not shown). Further studies will be needed to determine the mechanism by which TM regulates p38 signaling. In our study, we have begun to investigate the clinical implications of TM in mediating p38 activation. In Fig. 6, we demonstrated that the potential of TM treatment to enhance chemotherapy response using short-term cisplatin exposure and combinatorial treatment of doxorubicin and cisplatin. Moreover, our results suggest that TM may also be effective at reversing cisplatin-resistance, as demonstrated by enhanced p38 activation and cell death of A2780CIS cells in response to TM and cisplatin combinatorial treatment. Importantly, a TM-induced increase in cisplatin sensitivity occurs in a variety of gynecological cancer cell types (Fig. 6C), suggesting our results are not limited to ECC-1 cells. Future studies will determine if the mechanism by which TM potentiates cisplatin-induced cytotoxicity in other cancer cells is comparable to what we have observed in ECC-1 cells.

To conclude, our findings show that TM enhances cisplatin sensitivity in gynecological cancer cells. TM pretreatment blocks cisplatin-mediated EGFR tyrosine phosphorylation, yet potentiates p38 activation and receptor serine phosphorylation by cisplatin. We also found that p38 activation is involved in cisplatin-mediated EGFR degradation. Future studies will be needed to determine how p38 activation leads to receptor degradation, and how TM pretreatment mediates site-specific modulations of receptor phosphorylation after cisplatin exposure. Nevertheless, our results suggest that TM as an adjuvant to cytotoxic therapy may possess a therapeutic benefit due to inhibition of EGFR activation and promotion of EGFR degradation, which appears to occur in part by augmentation of cytotoxic p38 signaling pathways. Our findings may help create a foundation to better understand and improve current anti-EGFR therapies for the treatment of cancer.

## Materials

### Cell lines, cell culture, and reagents

ECC-1 (human endometrial) and SiHa (human cervical) cancer cell lines were purchased from the American Type Culture Collection and the A2780CIS (human ovarian) cancer cell line was supplied by Sigma Aldrich. A2780 (human ovarian) cancer cells were provided by Dr. Alexander Brodsky (Brown University). ECC-1 cells were grown in RPMI-1640 medium. SiHa, A2780, and A2780CIS cell lines were grown in Dulbecco's modified eagle medium. All culture media was supplemented with 10% fetal calf serum or bovine calf serum, penicillin (100 units/mL), and streptomycin (100 μg/mL). Cells were cultured at 37 °C with 5% CO_2_ in a humidified incubator. Reagents were purchased as follows: TM (as in ammonium salt), SRB, cisplatin, doxorubicin, anisomycin, cycloheximide (Sigma Aldrich); BIRB-796 (Selleck); AG1478 (Cell Signaling Technology); Recombinant human EGF (Invitrogen); cleaved PARP, cleaved caspase-3, SOD1, p-ERK, ERK, p38, p-p38, p-EGFR (Y1068) antibodies (Cell Signaling Technology); p-EGFR (S1046/7) antibody (Epitomics); GAPDH antibody (Santa Cruz Biotechnology). Cisplatin stock solution (1.67 mg/ml) was prepared in 0.9% sodium chloride solution.

### Cell proliferation, viability, apoptosis detection assay

Sulforhodamine B (SRB) cell proliferation, MTS cell viability, and TUNEL and Caspase-3/7 apoptosis detection assays were carried out following the protocols as described previously^30^,^44^,^45^.

### SOD activity assay

To determine SOD activity we used SOD Detection Kit (Cell Technology), which utilizes the reduction of tetrazolium into a water-soluble formazan dye in the reaction with superoxide generated by xanthine oxide activity. ECC-1 cells were treated with vehicle or TM (15 μM) for 20 h. Protein concentration in cell lysates was measured using Bio-Rad DC Protein Assay Kit (BioRad). Equal amounts of protein were subjected to SOD activity assay following the manufacturer’s protocol. Absorbance was measured at 450 nm using Multiskan RC microplate reader (Thermo).

### Transfection with siRNA

The following siRNA products from Thermo Scientific were used: control siRNA (siGENOME Non-Targeting siRNA) and human SOD1 siRNA (siGENOME SMARTpool, Target Sequence: UCGUUUGGCUUGUGGUGUA, ACAAAGAUGGUGUGGCCGA, GUGCAGGGCAUCAUCAAUU, UUAAUCCUCUAUCCAGAAA). ECC-1 cells were transfected with siRNA (final concentration: 50 nM) using lipofectamine 2000 (Invitrogen). 5 μl of lipofectamine, incubated in 250 μl of Opti-MEM (Invitrogen) for 5 min, was combined with the equal volume of Opti-MEM containing siRNA. After 20 min incubation the mixture was added to cells contained in 2 ml of antibiotic-free media, which was replaced with fresh medium after 7 h incubation.

### Immunoblotting

Cells were washed twice with phosphate buffered saline and lysed with Cell Lysis Buffer (Cell Signaling Technology) supplemented with 1 mM PMSF. The protein concentration of the lysate was measured using Bio-Rad DC Protein Assay Kit (BioRad). The samples were incubated in NuPAGE LDS sample buffer and Sample Reducing Agent (Invitrogen) at 70 °C for 10 min. Proteins were separated by NuPAGE Gel system (Invitrogen) using a 4–12% Tris-Bis precast gel and MES SDS running buffer, transferred onto a PVDF membrane (BioRad), blocked with 5% nonfat dry milk (BioRad) in TBS-Tween 20 (0.1%) buffer, and probed using respective antibodies indicated for experiments. The blots were visualized by ECL Prime Western Blotting Detection Reagents (GE Healthcare). The membrane was stripped using OneMinute Advance Western Blot Stripping buffer (GM Biosciences) and re-blocked before being re-probed with other antibodies. Densitometry was measured by Image J software.

## Additional Information

**How to cite this article**: Kim, K. K. *et al.* Tetrathiomolybdate mediates cisplatin-induced p38 signaling and EGFR degradation and enhances response to cisplatin therapy in gynecologic cancers. *Sci. Rep.* **5**, 15911; doi: 10.1038/srep15911 (2015).

## Supplementary Material

Supplementary Information
