# Supplementary material for: Tetrathiomolybdate mediates cisplatin-induced p38 signaling and EGFR degradation and enhances response to cisplatin therapy in gynecologic cancers
Source: Sci Rep. 2015 Nov 16;5:15911. doi: 10.1038/srep15911 (PMC4644948; doi:10.1038/srep15911)
Supplement: Supplementary Information [file srep15911-s1.doc]

**Supplementary Information**

**Tetrathiomolybdate mediates cisplatin-induced p38 signaling and EGFR degradation and enhances response to cisplatin therapy in gynecologic cancers**

Kyu Kwang Kim, Alex Han, Naohiro Yano, Jennifer R. Ribeiro, Elizabeth Lokich, Rakesh K. Singh, Richard G. Moore

Molecular Therapeutics Laboratory, Program in Women's Oncology, Departments of Obstetrics and Gynecology, Women and Infants Hospital, Alpert Medical School, Brown University, Providence, RI, USA

E-mail: [kkim@wihri.org](mailto:kkim@wihri.org)

**
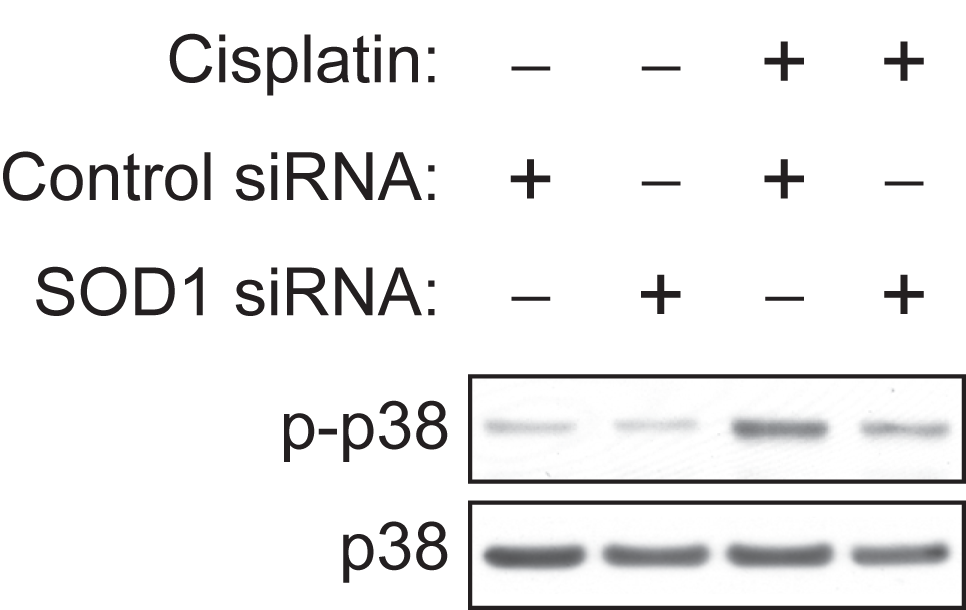
**

**Figure S1:** ECC-1 cells were transfected for 42 h with 50 nM siRNA pool against SOD1 or with non-targeting control, after which the cells were treated with or without cisplatin (30 µM) for 3 h. Immunoblotting was performed for p-p38 or p38.
